# Bioassay-guided isolation of three new alkaloids from *Suillus bovinus* and preliminary mechanism against ginseng root rot

**DOI:** 10.3389/fmicb.2024.1408013

**Published:** 2024-05-02

**Authors:** Miaomiao Xiong, Xiaomin Yang, Lan Yao, Zhuang Li, Jinxiu Zhang, Jianhua Lv

**Affiliations:** ^1^College of Life Sciences, Hebei Normal University, Shijiazhuang, China; ^2^Institute of Biology, Hebei Academy of Science, Shijiazhuang, China

**Keywords:** *Fusarium solani*, *Suillus bovinus*, mushrooms, alkaloids, antifungal activities

## Abstract

In order to control the occurrence of ginseng root rot caused by *Fusarium solani* (Mart.) Sacc., the antifungal compounds of the mushroom *Suillus bovinus* were investigated. And three new alkaloids (**1**–**3**), named bovinalkaloid A–C, along with one known analog (**4**), were isolated and identified by bioassay-guided isolation and spectroscopic analyses. Compound **1** strongly inhibited the mycelial growth and spore germination of *F. solani* with minimum inhibitory concentration of 2.08 mM. Increases in electrical conductivity, nucleic acid, and protein contents, and decreases in lipid content showed that the membrane permeability and integrity were damaged by compound **1**. Compound **1** also increased the contents of malondialdehyde and hydrogen peroxide and the activities of antioxidant enzymes, indicating that lipid peroxidation had taken place in *F. solani*. Compound **1** may serve as a natural alternative to synthetic fungicides for the control of ginseng root rot.

## 1 Introduction

*Panax ginseng* C. A. Mey is a deciduous perennial herb with important medicinal and economic values (Xu et al., 2017). It is mainly distributed in the northeastern regions of China and Korea and the eastern region of Siberia (Yu et al., 2018). For thousands of years, ginseng has been widely cultivated for its medicinal value (Xiao et al., 2016). Ginseng root rot was caused by *Fusarium* spp., *Fusarium solani* was one of the most important pathogens that can reduce the yield and quality of ginseng, thereby seriously constraining the economic development of ginseng (Li et al., 2020). Currently, the disease is controlled by applying synthetic fungicides and chemical products. However, the indiscriminate use of chemical fungicides causes serious health issues, environmental pollution, and development of resistance. Therefore, it is imperative to develop new safe and effective fungicides based on natural metabolites.

Mushrooms are notable for their large and visible fruiting bodies. They are consumed for their nutritional and medicinal values (Wang et al., 2013). Besides their high contents of proteins, dietary fiber, vitamins, and minerals, and low lipid content, mushrooms also contain a variety of secondary metabolites, such as fatty acids, triterpenoids, alkaloids, sterols, nucleosides, and phenolic compounds (Wang et al., 2017). To date, several compounds isolated from mushrooms have been demonstrated to exhibit effective antifungal activity. For example, a novel peptide isolated from the fruiting bodies of the mushroom *Pleurotus eryngii* can inhibit mycelial growth of *Mycosphaerella arachidicola* and *Fusarium oxysporum* (Wang and Ng, 2004). A furanone derivative isolated from the edible mushroom *Grifola frondosa* have a broad spectrum of antifungal activity against the plant pathogens (He et al., 2016). A nerolidol mannoside isolated from the *Schizophyllum commune* displayed antifungal activities against the plant pathogenic fungi *Botrytis cinerea*, *Rhizoctonia*, and *Alternaria solani* (Woo et al., 2019).

*Suillus bovinus*, an edible mushroom belonging to the *Suillus* genus, is broadly distributed across various regions globally (Feng et al., 2022). In recent years, research about *S. bovinus* focus mainly on the mechanisms related to symbiotic interactions with trees (Sun et al., 2023). Additionally, the pharmacological studies indicated that *S. bovinus* possessed the effect of inhibiting CYP enzymes (Huang et al., 2009). However, there are no reports concerning the antifungal activity of *S. bovinus*. In this study, we found that the extracts of *S. bovinus* exhibited good antifungal activity against *F. solani*. Subsequently, using a bioassay-guided separation procedure to explore the antifungal compounds from *S. bovinus* and to elucidate the possible antifungal mechanism. This will provide an alternative method of control for ginseng root rot and provide a theoretical foundation for the disease management of ginseng.

## 2 Materials and methods

### 2.1 Instruments and chemicals

NMR spectra were obtained using a Bruker DRX-600 spectrometer. HR-ESI-MS were recorded on an AB SCIE x500 Q-TOF MS spectrometer (AB Sciex Pte. Ltd., MA, United States). High-performance liquid chromatography (HPLC) was carried out using a Waters2535 chromatography system (Waters, Milford, MA, United States) equipped with a Waters 2,489 UV/visible detector with a YMC-Pack ODS-A (250 × 10 mm, 5 μm) column (YMC Co., Ltd., Kyoto, Japan). Acetonitrile and water (HPLC grade) were purchased from Fisher Scientific Ltd.

### 2.2 Mushroom material and pathogenic fungal strain

The fresh fruiting bodies of *S. bovinus* were collected from Chengde City, Hebei Province, China, in September 2022 and identified based on morphological characteristic and molecular analysis. The pathogenic fungus *F. solani* was provided by Professor Jie Gao (College of Plant Protection, Jilin Agricultural University, Changchun, China).

### 2.3 Extraction and bioassay-guided isolation

The fruiting bodies of *S. bovinus* were subjected to air-drying for 48 h followed by extraction using 95% ethanol (60 L, three times) for 24 h to obtain the crude extract weighing 2,011 g. The crude extract was suspended in 3 L of water and then sequentially partitioned with petroleum ether (3 × 3 L, 2 h each), ethyl acetate (3× 3 L, 2 h each), and n-butanol (3 × 3 L, 2 h each), to acquire a petroleum ether extract (PE, 422.2 g), an ethyl acetate extract (EA, 299.5 g), an n-butanol extract (BT, 554.3 g) and a remainder water extract (WT, 681.8 g). Antifungal assay revealed that EA showed higher activity and was selected for further isolation. Then, the EA was separated by silica gel column chromatography (200–300 mesh), eluted with a CH_2_Cl_2_/MeOH gradient (50:1–0:1), providing 10 fractions (F1–F10). The F6 which showed the best antifungal activity was further separated by Sephadex LH-20 (methanol) to obtain eight subfractions (F6.1–F6.8). The F6.4 and F6.6 exhibited much better antifungal activity than other subfractions. The F6.4 was further purified by semi-preparative RP-HPLC (YMC ODS-A column, 250 × 10 mm, 5 μm, 2.5 mL/min, acetonitrile/water, 20:80) to obtain compounds **1** (32.5 mg, *t*_R_ = 20.5 min), **2** (27.2 mg, *t*_R_ = 22.5 min), and **4** (19.6 mg, *t*_R_ = 26.1 min). The F6.6 was further purified by semi-preparative RP-HPLC (2.5 mL/min, acetonitrile/water, 25:75) to obtain compound **3** (21.2 mg, *t*_R_ = 23.5 min).

### 2.4 Inhibition of mycelial growth

The mycelial growth inhibition assay was performed as previously described (Palacios et al., 2014; Bhutia et al., 2016), with minor modification. The samples (extracts and compounds) were diluted with pure water. Under aseptic conditions, the solution was filtered through a 0.22 μm organic filter to obtain the sterile solution. The solution was then added to sterilized PDA medium. Afterward, a seven-day-old mycelial disk (6 mm) was obtained from the periphery of a culture of *F. solani* and was placed in the center of the PDA culture dish. Media without samples were used as controls, and each treatment was repeated three times and incubated in the dark at 25°C. Colony diameters were measured by the cross method using a Vernier caliper after 8 days of incubation. The mycelial growth inhibition rate was calculated according to the following formula:


$$\mathrm{Growth}\;\mathrm{inhibition}\;\mathrm{rate}\;\left(\%\right)=\left(D_{c}-D_{t}\right)/\left(D_{c}-D_{i}\right)\times 100$$

Where D_c_ and D_t_ are the colony diameter of the control group and treated group, respectively; D_i_ is the initial colony diameter.

The minimal inhibitory concentration (MIC) was defined as the lowest concentration that inhibited visible growth of *F. solani* after 2 days of incubation in the dark at 25°C (Zhang et al., 2020).

### 2.5 Inhibition of spore germination

The spore germination inhibition assay was performed as previously described (Chowdhury and Bae, 2018; Elsherbiny et al., 2021), with minor modification. Briefly, the tested *F. solani* strain was cultured in PDA plate medium at 25°C for 10 days. The plates were washed with 5 mL sterile water to acquire a spore suspension (1 × 10^6^ spores/mL). Spore suspensions (10 μL, conidia) with different concentrations of compound **1** were incubated at 25°C for 24 h. The control group was given an equal amount of sterile water. Approximately 100 spores were observed with an inverted microscope (40 × magnification). Each treatment was repeated three times. Spores were germinated when the length of the germ tube was longer than the diameter of the spore. The spore germination inhibition rate was calculated according to the following formula:


$$\mathrm{Inhibition}\;\mathrm{rate}\;\left(\%\right)=\left(G_{c}-G_{t}\right)/G_{c}\times 100$$

Where G_c_ and G_t_ are the number of germinated spores of the control group and treated group, respectively.

### 2.6 Observation of the mycelial morphology of *Fusarium solani*

Four cover glasses were inserted on a slant into the PDA medium. Simultaneously, a seven-day-old mycelial disk (6 mm) was obtained from the periphery of the culture of *F. solani* and was placed in the center of the PDA culture dish. When the mycelium grew to two-thirds of the area of the cover glass, cover glasses were removed, and slides were immersed in PDA medium containing compound **1** (MIC) for 24 h at 25°C. Media without compound **1** were used as controls (Chen et al., 2020). Mycelial morphology was observed under an optical microscope (Olympus CKX53, Tokyo, Japan).

### 2.7 Assessment of membrane permeability of *Fusarium solani*

Electrolyte leakage, nucleic acid content, and protein content were determined as previously described (Chen et al., 2020; Souza et al., 2020), with slight modification. Compound **1** was added to the fungal suspension at a final concentration of MIC and incubated for 0, 2, 4, 6, 8, 10, 15, and 20 h. The control group was prepared without compound **1**. Subsequently, the suspension was centrifuged at 12,000 rpm for 3 min, and the supernatant was collected. The electrical conductivity was evaluated using a conductivity meter (SevenMulti, Shanghai Mettler Toledo Co., Ltd., Shanghai, China). Nucleic acid and protein contents were measured at wavelengths of 260 and 280 nm, respectively, using a Shimadzu UV-2600 spectrophotometer (Shimadzu UV-2600, Tokyo, Japan).

### 2.8 Determination of total lipid content

Total lipid content was determined as previously described (Helal et al., 2007), with minor modification. Compound **1** was added to the fungal suspension at a final concentration of MIC and incubated for 0, 2, 4, 6, 8, 10, 15, and 20 h Subsequently, the mycelia (1 g, dry weight) were extracted using a methanol-chloroform-water (2:1:1, v/v) mixture. Saline solution (0.9% NaCl, 0.2 mL) was added to the lower phase containing lipids, shaken, and then centrifuged at 5000 rpm for 5 min. The lower phase containing lipids was oven dried at 65°C to constant weight. After drying, 0.5 mL 12.4 mol/L H_2_SO_4_ was added to the extracted lipids, and the mixture was heated in a boiling water bath for 5 mins and left to cool down at room temperature. Then, 3 mL phosphovanillin was added and the mixture was shaken. The mixture was allowed to stand for 10 min at room temperature, and the absorbance at 520 nm was measured to determine total lipid content from a standard curve obtained using cholesterol.

### 2.9 Determination of contents of malondialdehyde and hydrogen peroxide

The malondialdehyde (MDA) and hydrogen peroxide (H_2_O_2_) contents were measured using commercial MDA and H_2_O_2_ assay kits (A003-1, A064-1 Jiancheng Bioengineering Institute, Nanjing, China) respectively.

### 2.10 Determination of activities of antioxidant enzymes

The catalase (CAT) activity, peroxidase (POD) activity, and superoxide dismutase (SOD) activity were measured using commercial CAT, POD, and SOD assay kits (A007-1, A084-3, A001-1, Jiancheng Bioengineering Institute, Nanjing, China), respectively.

### 2.11 Statistical analysis

Data were analyzed with Origin 8.5 software (OriginLab Corporation, Hampton, MA, United States), and all data were expressed as means ± standard deviation. Means were compared by Duncan’s new multiple range test. *p* < 0.05 was used to identify statistical significance.

## 3 Results and discussion

### 3.1 Extraction and bioassay-guided isolation of antifungal compounds

**Figure 1:**
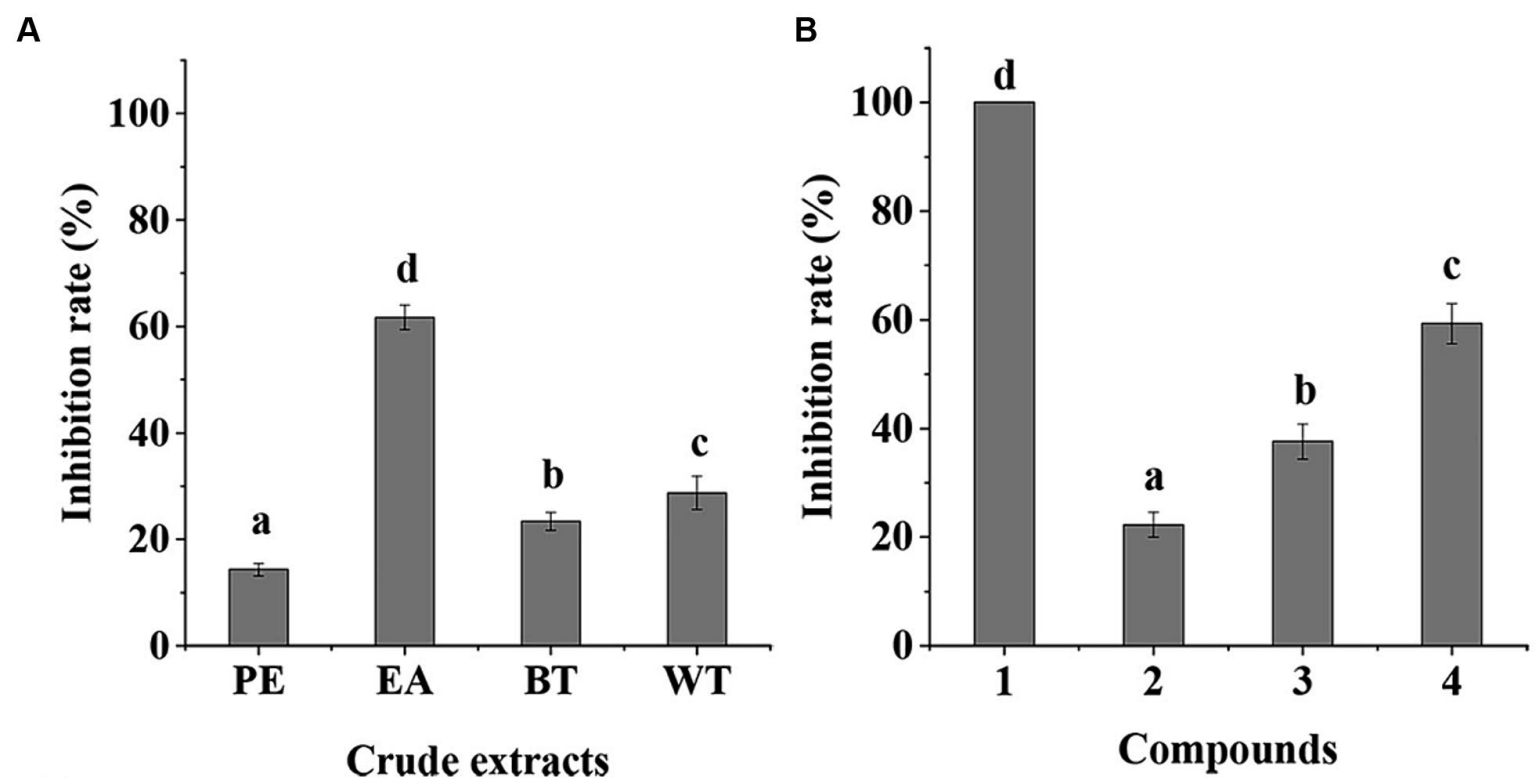
Antifungal activity of crude extracts **(A)**, and isolated compounds **(B)** from *Suillus bovinus*. The data were expressed as means ± standard deviation of mean. a, b, c, d indicates statistically significant differences (*p* < 0.05).

**Figure 2:**
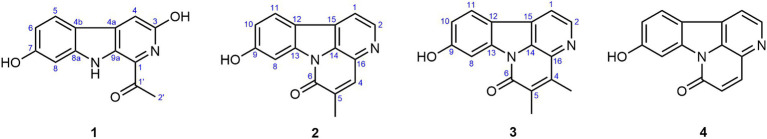
The chemical structures of compounds **1**–**4**.

To screen for the compounds with antifungal activity against *F. solani*, a 95% aqueous ethyl alcohol crude extract of *S. bovinus* was sequentially partitioned with petroleum ether, ethyl acetate, and n-butanol to yield four solvent-soluble fractions, PE, EA, BT, and WT. Then, the antifungal activities of the four fractions were tested at concentrations of 2 mg/mL. As shown in Figure 1A, the EA (61.72%) showed higher antifungal activity than PE (14.33%), BT (23.42%), and WT (28.71%). The results indicated the EA may contain the antifungal compounds. Afterward, bioassay-guided separation of EA led to the isolation of four compounds (**1**–**4**) (Figure 2).

### 3.2 Structure elucidation and antifungal activity of compounds **1**–**4**

**Figure 3:**
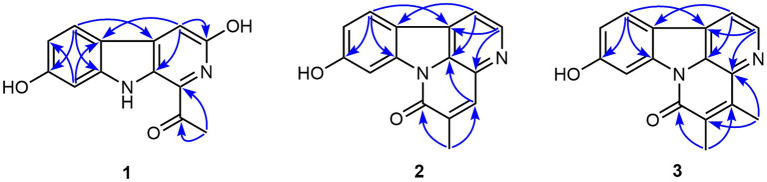
The significant HMBC correlations of compounds **1**–**3**.

Compound **1** possessed a molecular formula of C_13_H_10_N_2_O_3_ by the negative HR-ESI-MS (*m/z* 241.0619 C_13_H_9_N_2_O_3_^−^, calcd. 241.0619), implying 10 degrees of unsaturation. The ^1^H NMR spectrum (Table 1) in DMSO-*d*_6_ of **1** showed one exchangeable proton signal at *δ*_H_ 11.19 (1H, s, NH), three aromatic protons at *δ*_H_ 7.93 (1H, d, *J* = 8.5 Hz, H-5), 7.02 (1H, d, *J* = 1.9 Hz, H-8) and 6.64 (1H, dd, *J* = 8.5, 1.9 Hz, H-6) assignable to a 1,2,4-trisubstituted benzene ring, one isolated aromatic singlet at *δ*_H_ 7.46 (1H, s, H-4), as well as one methyl singlet at *δ*_H_ 2.68 (3H, s, H-2′). The ^13^C NMR spectrum (Table 1) showed one conjugated ketone carbon at *δ*_C_ 200.5 (s, C-1′), 11 *sp*^2^ carbon signals (4 × CH and 7 × C) in the range from 97.8—159.4 ppm, as well as one aliphatic methyl carbon at *δ*_C_ 25.9 (q, C-2′). The above NMR features were very similar to those of arenarine D (1-acetyl-7-hydroxy-β-carboline; Wu et al., 1989), and the signal differences were only from the pyridine ring moiety. In consideration of the molecular formula and the presence of an isolated aromatic singlet, a phenolic hydroxy group should be substituted at C-3 or C-4 of the pyridine ring. The observable HMBC correlations (Figure 3) from the aromatic singlet at *δ*_H_ 7.46 (1H, s) to C-4b [*δ*_C_ 112.2 (s)] and C-9a [*δ*_C_ 131.2 (s)] revealed the existence of a phenolic hydroxy group at C-3. The resulting structure was further verified by the detailed 2D NMR analysis. Therefore, the structure of **1** was established as 1-acetyl-3,7-dihydroxy-β-carboline and named as bovinalkaloid A, shown in Figure 2.

**Table 1:** The ^1^H and ^13^C NMR data for 1 in DMSO-*d*_6_.

No.	^1^H NMR	^13^C NMR
1	―	130.2 (s)
3	―	155.1 (s)
4	7.46 (1H, s)	103.8 (d)
4a	―	136.4 (s)
4b	―	112.2 (s)
5	7.93 (1H, d, 8.5)	123.0 (d)
6	6.64 (1H, dd, 8.5, 1.9)	109.6 (d)
7	―	159.4 (s)
8	7.02 (1H, d, 1.9)	97.8 (d)
8a	―	145.5 (s)
9a	―	131.2 (s)
1′	―	200.5 (s)
2′	2.68 (3H, s)	25.9 (q)
NH	11.19 (1H, s)	―

Compound **2**, obtained as yellow amorphous powder, had a molecular formula of C_15_H_10_N_2_O_2_ by the negative HR-ESI-MS (*m/z* 249.0669 C_15_H_9_N_2_O_2_^−^, calcd. 249.0670), indicating 12 indices of hydrogen deficiency. The ^1^H NMR spectrum (Table 2) in DMSO-*d*_6_ of **2** exhibited one phenolic hydroxy signal at *δ*_H_ 10.51 (1H, br s), a pair of mutually coupled heterocyclic aromatic doublets at *δ*_H_ 8.71 (1H, d, *J* = 5.0 Hz, H-2) and 8.07 (1H, d, *J* = 5.0 Hz, H-1), three aromatic protons at *δ*_H_ 8.15 (1H, d, *J* = 8.5 Hz, H-11), 8.01 (1H, d, *J* = 2.1 Hz, H-8) and 6.99 (1H, dd, *J* = 8.5, 2.1 Hz, H-10) assignable to a 1,2,4-trisubstituted benzene ring, one isolated olefinic or aromatic signal at *δ*_H_ 8.02 (1H, br s, H-4), as well as one olefinic methyl singlet at *δ*_H_ 2.31 (3H, br s) in the up-field region. The ^13^C NMR spectrum (Table 2) showed a total of 15 carbon resonances, including 14 *sp*^2^ carbon signals (6 × CH and 8 × C) in the range from 103.1—160.5 ppm, as well as one aliphatic methyl carbon at *δ*_C_ 17.4 (q). The aforementioned NMR features and its characteristic UV curve suggested that **2** should be a canthin-6-one alkaloid, structurally similar to 9-hydroxycanthin-6-one (Leonardus et al., 1991). A careful comparison of the NMR data of **2** with those of 9-hydroxycanthin-6-one revealed that the signal differences were only from the lactam ring moiety. According to the observable HMBC correlations (Figure 3) from the methyl proton signal to C-4 [*δ*_C_ 135.9 (d)] and C-6 [*δ*_C_ 159.8 (s)], and from H-4 [*δ*_H_ 8.02 (1H, br s)] to C-14 [*δ*_C_ 130.9 (s)], the newly emerging aliphatic methyl group was positioned at C-5. The resulting structure was further rechecked by the detailed 2D NMR analysis. Therefore, the structure of **2** was established as 9-hydroxy-5-methylcanthin-6-one and named as bovinalkaloid B, shown in Figure 2.

**Table 2:** The ^1^H and ^13^C NMR spectral data for compounds **2** and **3** in DMSO-*d*_6_.

No.	**2**		**3**	
	^1^H NMR	^13^C NMR	^1^H NMR	^13^C NMR
1	8.07 (1H, d, 5.0)	115.1 (d)	8.09 (1H, d, 5.0)	115.3 (d)
2	8.71 (1H, d, 5.0)	145.8 (d)	8.74 (1H, d, 5.0)	145.2 (d)
4	8.02 (1H, br s)	135.9 (d)	―	144.3 (s)
5	―	137.1 (s)	―	132.3 (s)
6	―	159.8 (s)	―	159.4 (s)
8	8.01 (1H, d, 2.1)	103.1 (d)	8.01 (1H, d, 2.1)	103.0 (d)
9	―	160.5 (s)	―	160.4 (s)
10	6.99 (1H, dd, 8.5, 2.1)	114.0 (d)	6.98 (1H, dd, 8.5, 2.1)	113.8 (d)
11	8.15 (1H, d, 8.5)	124.7 (d)	8.13 (1H, d, 8.5)	124.5 (d)
12	―	116.0 (s)	―	115.9 (s)
13	―	140.7 (s)	―	140.7 (s)
14	―	130.9 (s)	―	129.8 (s)
15	―	129.5 (s)	―	129.6 (s)
16	―	135.4 (s)	―	135.7 (s)
4-CH_3_	―	―	2.60 (3H, s)	12.9 (q)
5-CH_3_	2.31 (3H, br s)	17.4 (q)	2.27 (3H, s)	13.3 (q)

Compound **3**, yellow amorphous powder, possessed a molecular formula of C_16_H_12_N_2_O_2_ by the negative HR-ESI-MS (*m/z* 263.0821 C_16_H_11_N_2_O_2_^−^, calcd. 263.0826), which was 14 Da (CH_2_) more than that of **2**. The ^1^H NMR spectrum (Table 2) in DMSO-*d*_6_ of **3** also showed a pair of characteristic heterocyclic aromatic doublets at *δ*_H_ 8.74 (1H, d, *J* = 5.0 Hz, H-2) and 8.09 (1H, d, *J* = 5.0 Hz, H-1), three aromatic protons assignable to a 1,2,4-trisubstituted benzene ring, as well as two olefinic methyl singlets in the up-field region. The ^13^C NMR spectrum (Table 2) showed 14 *sp*^2^ carbon signals (5 × CH and 9 × C) in the range from 103.0—160.4 ppm, as well as two methyl carbons at *δ*_C_ 13.3 (q) and 12.9 (q). The above NMR features were very similar to those of **2**, and allowed us to infer that the structure **3** should be a 4,5-dimethylated derivative of 9-hydroxycanthin-6-one. The HMBC correlations (Figure 3) from the methyl proton signal at *δ*_H_ 2.60 (3H, s) to C-5 [*δ*_C_ 132.3 (s)] and C-16 [*δ*_C_ 135.7 (s)], and from the other methyl signal at *δ*_H_ 2.27 (3H, s) to C-4 [*δ*_C_ 144.3 (s)] and C-6 [*δ*_C_ 159.4 (s)] confirmed the two methyl groups at C-4 and C-5, respectively. Thus, the structure of **3** was established as 9-hydroxy-4,5-dimethylcanthin-6-one and named as bovinalkaloid A, shown in Figure 2.

By comparing with data reported in the literature, the known compound **4** was identified as 9-hydroxycanthin-6-one (Jiang and Zhou, 2008). All of the isolated compounds (**1**–**4**) were evaluated for their *in vitro* antifungal activity against *F. solani* (Figure 1B). At the concentration of 1 mg/mL, the compounds **1**–**4** exhibited some degree of antifungal activity, with inhibition rates of 100.00%, 22.31%, 37.59%, and 59.29%, respectively. Compound **1** had significant antifungal activity against *F. solani*. For a better understanding of the antifungal activity and mechanisms of compound **1**, a further in-depth study was required.

### 3.3 Mycelial growth and spore germination

**Figure 4:**
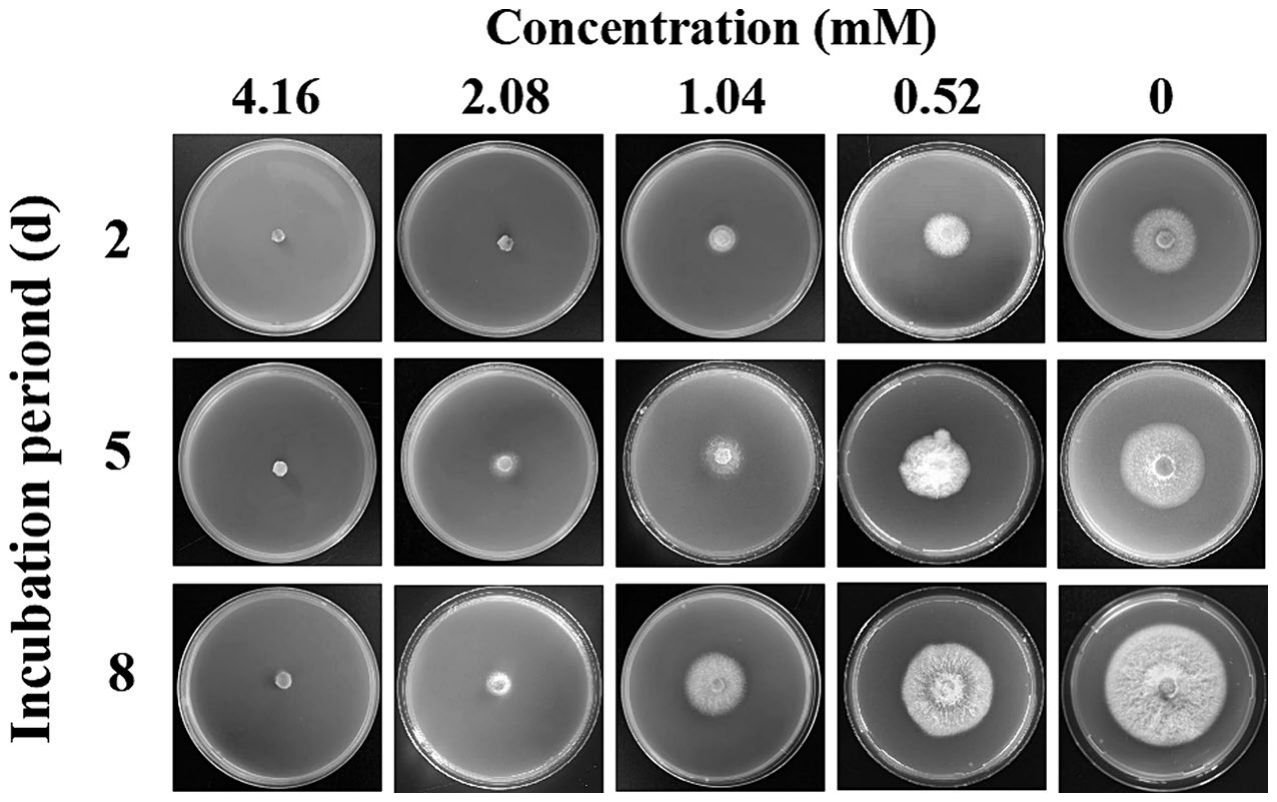
The inhibitory effect of different concentrations of compound **1** against the mycelial growth of *Fusarium solani.*

Compound **1** showed excellent antifungal activity against *F. solani* (Table 3). The inhibitory effect increased with increasing concentration. For compound **1** at a comparatively low concentration of 0.52 mM, the mycelial growth inhibition rate was 42.11%. When the concentration reached 1.04 mM, the mycelial growth inhibition rate was greater than 70%. In particular, the mycelial growth of *F*. *solani* on PDA medium was entirely inhibited by compound **1** at the concentration of 4.16 mM. The MIC of compound **1** are illustrated in Figure 4. Compound **1** at 2.08 mM or higher concentrations entirely inhibited visible growth of *F*. *solani* after 2 days of incubation in the dark at 25°C. Thus, the MIC of compound **1** was 2.08 mM.

**Table 3:** Effect of different concentrations of compound **1** on mycelial growth and spore germination of *Fusarium solani*.

Concentration (mM)	Growth inhibition rate (%)	Germination inhibition rate (%)
0	0	0
0.52	42.11 ± 2.33a	47.17 ± 4.12 a
1.04	70.19 ± 1.98 b	71.22 ± 3.14 b
2.08	96.21 ± 2.66 c	86.55 ± 2.81 c
4.16	100 ± 0.00 d	94.37 ± 3.22 d

Values are shown as mean ± standard deviation of mean (*n* = 3). a, b, c, d indicates significant differences according to Duncan’s test (*p* < 0.05).

We also researched the effect of compound **1** on the germination of *F*. *solani* spores (Table 3). The spore germination was significantly inhibited by compound **1** in a dose-dependent manner. When the concentration at 4.16 mM, the spore germination inhibition rate was 94.37%, less than 10% of *F*. *solani* spores germinated.

### 3.4 Effects of compound **1** on mycelial morphology

**Figure 5:**
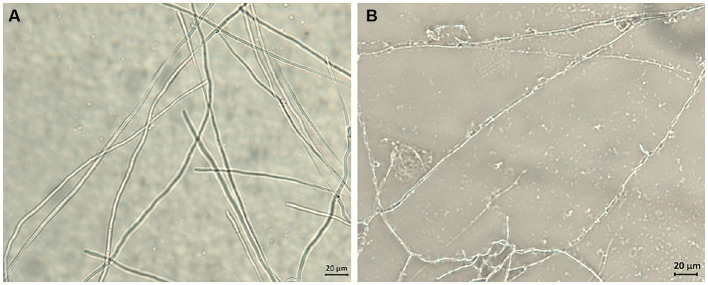
The effect of compound **1** on mycelial morphology of *Fusarium solani*. Normal mycelial morphology **(A)**; Mycelia treated with MIC of compound **1 (B)**.

The potential antifungal mechanisms of compound **1** against *F*. *solani* were investigated by light microscopy. Figure 5 shows the mycelial morphology in the control and compound **1** treated groups. The control *F*. *solani* mycelia were regular and complete, and the surface of the mycelium was smooth and without cracks (Figure 5A). However, the mycelia treated with compound **1** displayed a damaged morphology. As shown in Figure 5B, mycelia appeared collapsed, malformed, and atrophied, and their surface structure became fractured and rough.

Examination of the morphological changes in *F. solani* mycelia before and after being treated with compound **1** was important for discovering the targets upon which compound **1** act. Obviously, compound **1** treatment resulted in surface structure damage to *F*. *solani* mycelia. This implied that compound **1** might destroy the structure of the membrane system to change its permeability, inducing the leakage of intracellular substances. Similar research has demonstrated that with chelerythrine treatment, the mycelia of *Ustilaginoidea virens* collapse and become twisted, and the membrane becomes thin and rough (Wei et al., 2020). Based on the above analysis, we studied the effects of compound **1** on cell membrane permeability.

### 3.5 Effects of compound **1** on membrane permeability

**Figure 6:**
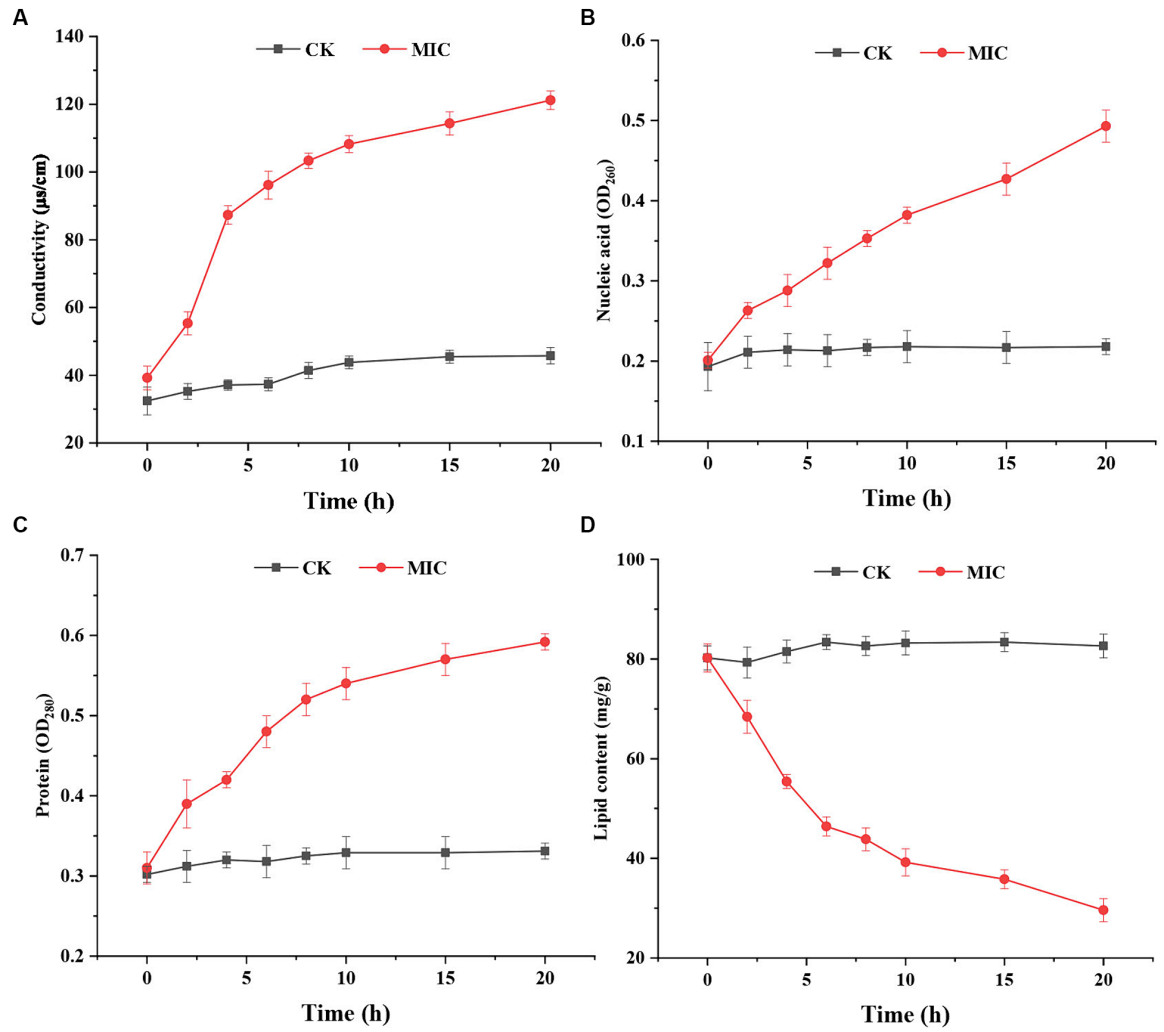
The effect of compound **1** on electrical conductivity **(A)**, nucleic acid content **(B)**, protein content **(C)**, and lipid content **(D)** of *Fusarium solani*.

The cell membrane is a semipermeable protective structure that can regulate the homeostasis of internal and external environments of fungi. When the membrane is stimulated by externally applied compounds, the electrical conductivity increases due to damage to cell membranes, consequently resulting in solute leakage (Cai et al., 2019). In the present study, electrolyte leakage, nucleic acid content, and protein content of the supernatant were used to determine the membrane permeability. The results (Figures 6A–C) indicated that after compound **1** treatment, the electric conductivity, nucleic acid content, and protein content were significantly higher than in the control group. After incubation for 20 h, the electric conductivity of the compound **1** treated group (121.24 μs/cm) was approximately 2.65-fold higher than that of the control group (45.78 μs/cm); the nucleic acid content (OD_260_) of the compound **1** treated group (0.493) was approximately 2.26-fold higher than that of the control group (0.218), and the protein content (OD_280_) of the compound **1** treated group (0.592) was approximately 1.79-fold higher than that of the control group (0.331).

These results further demonstrated that compound **1** might damage the cell membrane of *F*. *solani,* leading to leakage of the nucleic acids, proteins, and electrolytes. Similar results have been observed in *Botrytis cinerea* (Ji et al., 2020) and *Penicillium roqueforti* (Ju et al., 2020). After confirming that the membrane permeability of *F*. *solani* was affected by compound **1**, the main constituents of the membrane were measured. The integrity of the cell membrane is critical for *F*. *solani* growth.

### 3.6 Effects of compound **1** on total lipid content

Lipids are essential components of cell membranes, and they are vitally important for *F. solani* structural integrity (Lu et al., 2019). As shown in Figure 6D, the total lipid content of the control group remained largely the same at 80.23 ± 3.25 mg/g. In contrast, the lipid content of the compound **1** treated group was lower than that of the control group, and the content followed a gradually decreasing tend, reaching a minimum of 29.62 ± 2.89 mg/g at 20 h. The reduction of the total lipid content indicated the disruption of cell membrane integrity.

### 3.7 Effects of compound **1** on MDA and H_2_O_2_ content

**Figure 7:**
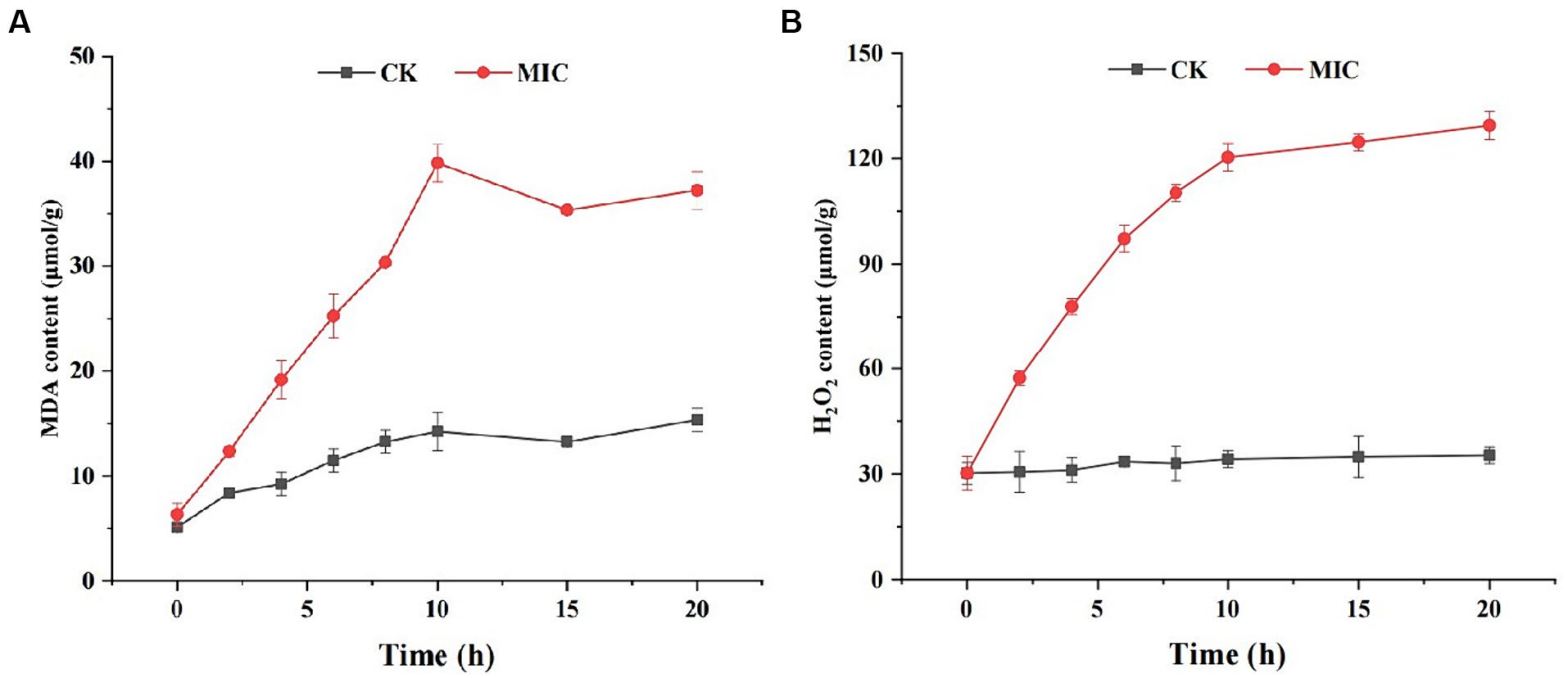
The effect of compound **1** on MDA **(A)** and H_2_O_2_ content **(B)** of *Fusarium solani*.

Numerous studies have reported that lipid peroxidation is one of the main causes of damage to cell membranes (Liu et al., 2020). MDA is a terminal product of lipid peroxidation, and it can cause the cross-linking polymerization of nucleic acids, proteins, and other biological macromolecules. We measured the content of MDA in mycelia of *F. solani* as an indicator of lipid peroxidation. As shown in Figure 7A, the MDA level of compound **1** treated group was consistently higher than that of the control group. Especially at 10 h, the MDA content of compound **1** treated group (39.83 μmol/g) was approximately 2.79-fold higher than that of the control group (14.23 μmol/g), indicating that lipid peroxidation had taken place in *F. solani*.

Lipid peroxidation is generally caused by excess reactive oxygen species (ROS) such as superoxide and H_2_O_2_ that are necessary products of aerobic metabolism (Li et al., 2018). If the generated ROS are not scavenged in a timely manner, they can cause oxidative damage to lipids, proteins, and DNA. To examine this, we measured H_2_O_2_ levels in *F. solani* and found that the H_2_O_2_ content in the compound **1** treated group was markedly higher than in the control group (Figure 7B). These results suggest that compound **1** could induce ROS generation and increase lipid peroxidation.

### 3.8 Effects of compound **1** on antioxidant enzymes activities

**Figure 8:**
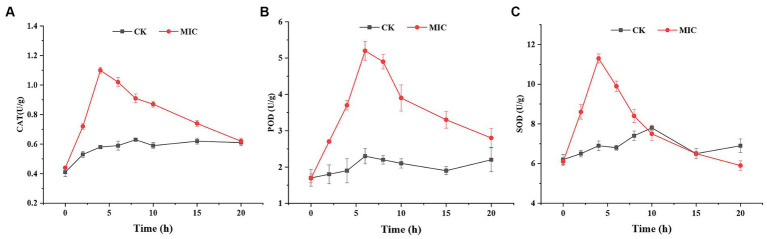
The effect of compound **1** on CAT activity **(A)**, POD activity **(B)**, and SOD activity **(C)** of *Fusarium solani*.

ROS production and scavenging maintain a dynamic balance in the normal cellular systems. Cells normally protect themselves against ROS damage by activating antioxidant defense systems (Li et al., 2012). CAT, POD, and SOD play crucial roles in antioxidant systems that scavenge ROS by transforming H_2_O_2_ into water (Yang et al., 2020). Therefore, it was necessary to measure the respective antioxidant enzyme activities of *F*. *solani* mycelia. As shown in Figure 8, CAT, POD, and SOD activities of the control group remained essentially unchanged during 0–20 h, while the antioxidant enzymes activities in the compound **1** treated group exhibited an initial increasing trend and then decreased.

Based on the results, it was supposed that in the early stages of compound **1** treatment, the defense system of *F. solani* up-regulated the levels of these antioxidant enzymes in response to increased ROS. However, with increasing compound **1** treatment time, the production of ROS overwhelmed the scavenging capacity of antioxidant enzymes, thereby disrupting the balance of ROS production and scavenging in *F. solani*. Immediately after, the antioxidant enzymes were damaged by excessive ROS, leading to a reduction in the activities of antioxidant enzymes and incrementing production of ROS. The accumulated ROS reacted with polyunsaturated fatty acids to produce MDA (lipid peroxidation) and eventually led to cell death. This further confirmed that compound **1** could induce ROS generation that caused lipid peroxidation, eventually resulting in cell membrane damage.

## 4 Conclusion

In the present study, four alkaloids including three new compounds (**1**–**3**), were isolated and identified by bioassay-guided isolation and spectroscopic analyses from mushroom *S. bovinus*. Compound **1** strongly inhibited the mycelial growth and spore germination of *F. solani*, the causal fungus of ginseng root rot. Furthermore, we investigated the antifungal mechanisms of compound **1**. Compound **1** could induce ROS generation that causes lipid peroxidation, resulting in cell membrane injury, solute and electrolyte leakage, and subsequent cell death. We believe that these findings will provide a theoretical foundation for the application of compound **1** as a potential natural fungicide for the control of ginseng root rot.

## Data availability statement

The original contributions presented in the study are included in the article/Supplementary material, further inquiries can be directed to the corresponding authors.

## Author contributions

MX: Formal analysis, Investigation, Writing – original draft. XY: Investigation, Supervision, Validation, Writing – review & editing. LY: Investigation, Validation, Visualization, Writing – original draft. ZL: Data curation, Project administration, Software, Visualization, Writing – review & editing. JZ: Formal analysis, Funding acquisition, Investigation, Validation, Writing – original draft. JL: Conceptualization, Funding acquisition, Project administration, Resources, Writing – original draft.
