# Supplementary material for: Bioassay-guided isolation of three new alkaloids from Suillus bovinus and preliminary mechanism against ginseng root rot
Source: Front Microbiol. 2024 May 2;15:1408013. doi: 10.3389/fmicb.2024.1408013 (PMC11096550; doi:10.3389/fmicb.2024.1408013)
Supplement: Supplementary file 1 [file Data_Sheet_1.docx]

Supplementary Material

**Bioassay-guided Isolation of Three New Alkaloids from *Suillus bovinus* and Preliminary Mechanism against Ginseng Root Rot**

**Miaomiao Xiong^1^, Xiaomin Yang^1^, Lan Yao^2^, Zhuang Li^1^, Jinxiu Zhang^1*^, Jianhua Lv^1*^,**

^1^College of Life Sciences, Hebei Normal University, Shijiazhuang, China

^2^Institute of Biology, Hebei Academy of Science, Shijiazhuang, China

***Correspondence:**

Jian-Hua Lv

lvjianhua@hebtu.edu.cn

Jinxiu Zhang

xiudou882003@163.com

**List of Supplementary Material**

**Figure S1**. HR-ESI-MS of compound **1**

**Figure S2**. ^1^H-NMR spectrum of compound **1**

**Figure S3**. ^13^C-NMR spectrum of compound **1**

**Figure S4**. HSQC spectrum of compound **1**

**Figure S5**. HMBC spectrum of compound **1**

**Figure S6**. ^1^H-^1^H COSY spectrum of compound **1**

**Figure S7**. HR-ESI-MS of compound **2**

**Figure S8**. ^1^H-NMR spectrum of compound **2**

**Figure S9**. ^13^C-NMR spectrum of compound **2**

**Figure S10**. HSQC spectrum of compound **2**

**Figure S11**. HMBC spectrum of compound **2**

**Figure S12**. ^1^H-^1^H COSY spectrum of compound **2**

**Figure S13**. HR-ESI-MS of compound **3**

**Figure S14**. ^1^H-NMR spectrum of compound **3**

**Figure S15**. ^13^C-NMR spectrum of compound **3**

**Figure S16**. HSQC spectrum of compound **3**

**Figure S17**. HMBC spectrum of compound **3**

**Figure S18**. ^1^H-^1^H COSY spectrum of compound **3**

**Figure S1**. HR-ESI-MS of compound **1**


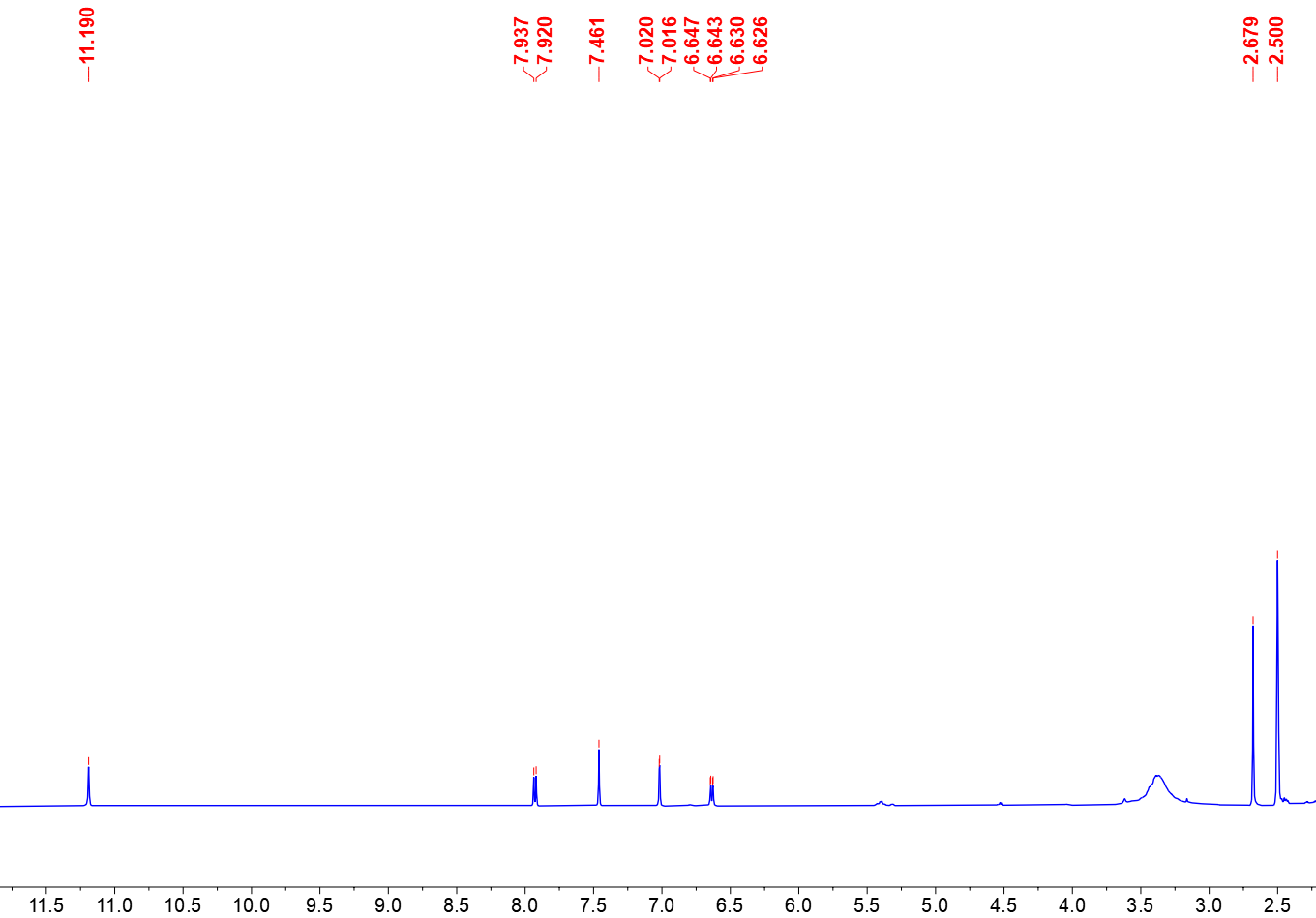


**Figure S2**. ^1^H-NMR spectrum of compound **1**


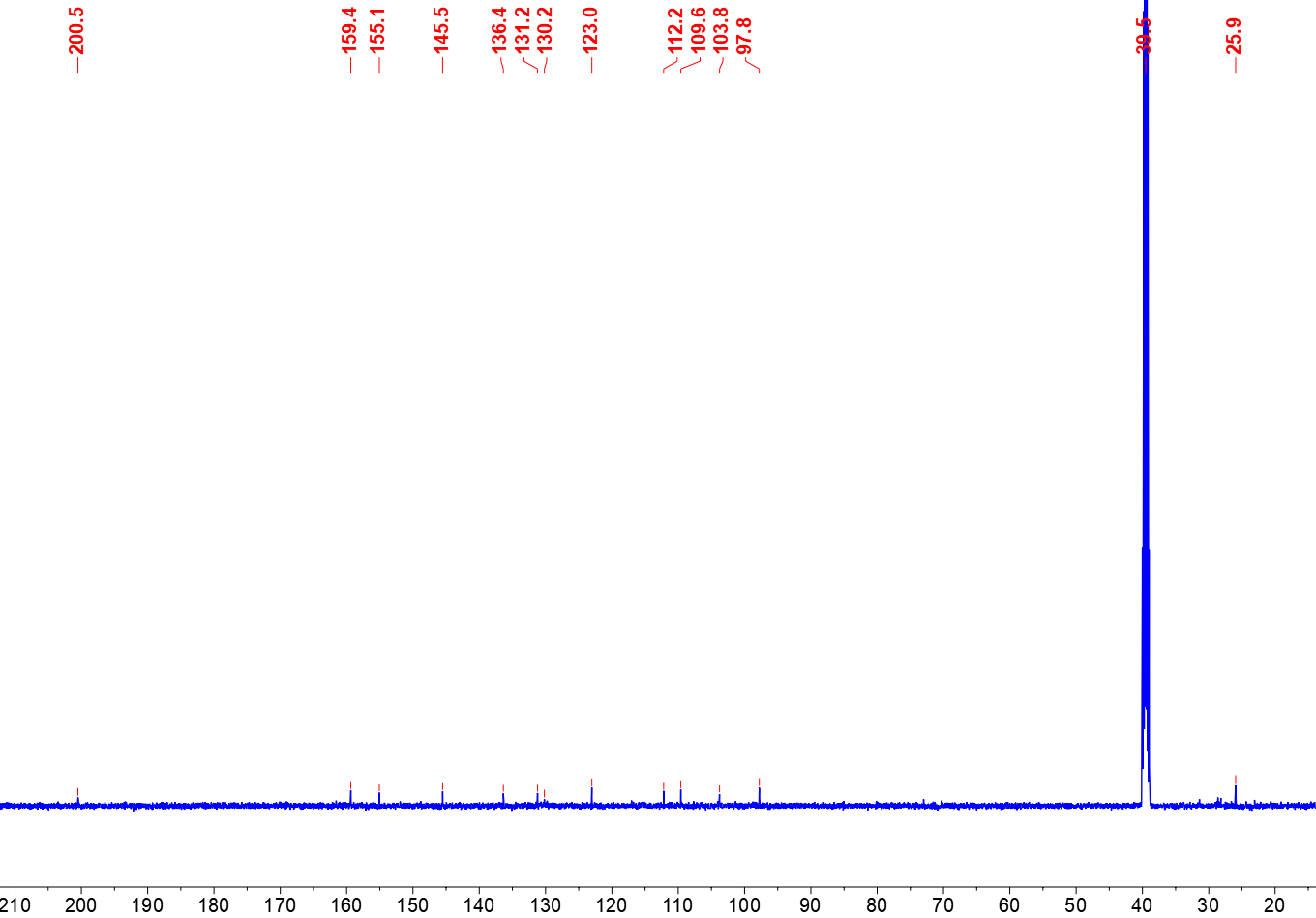


**Figure S3**. ^13^C-NMR spectrum of compound **1**


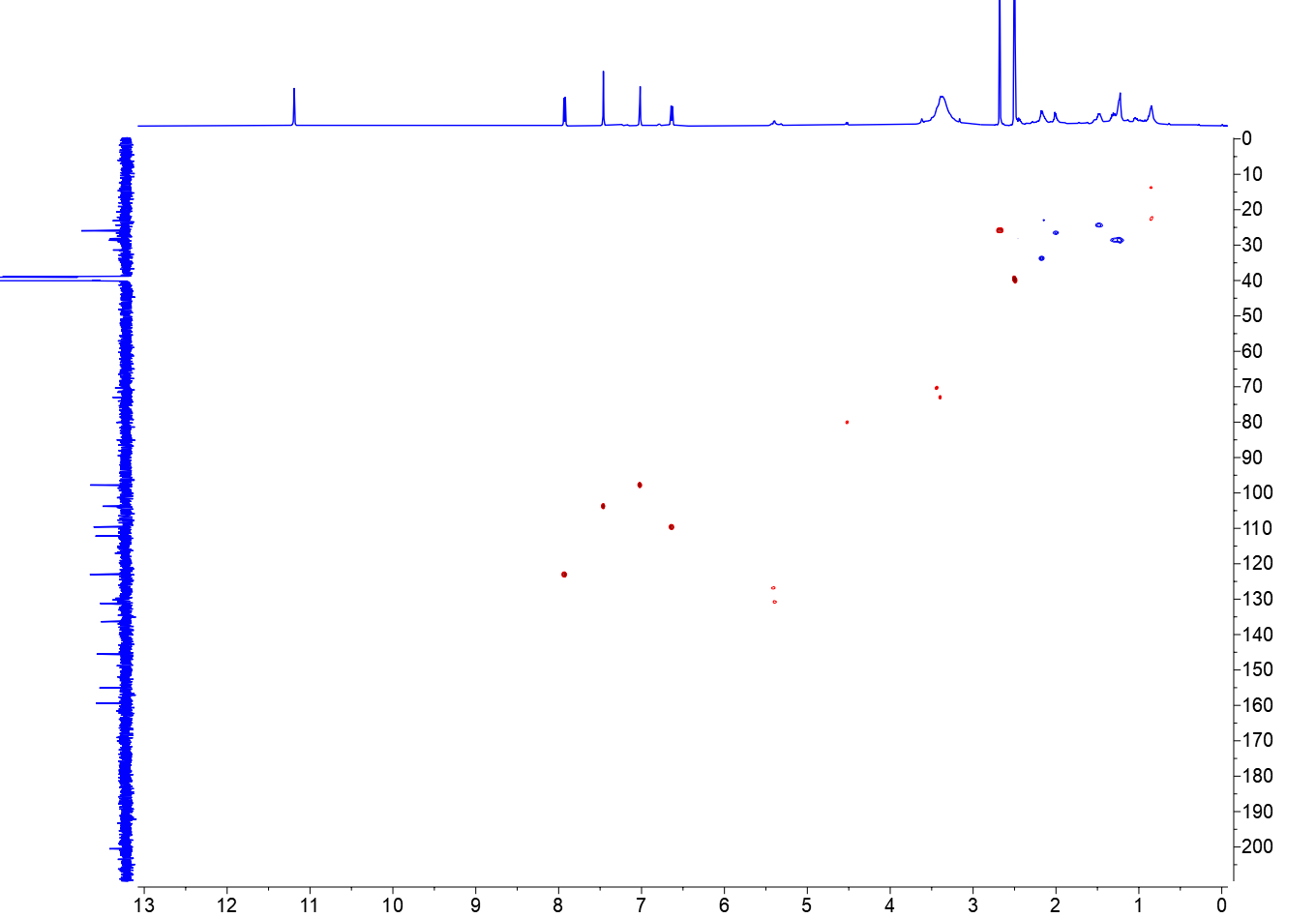


**Figure S4**. HSQC spectrum of compound **1**


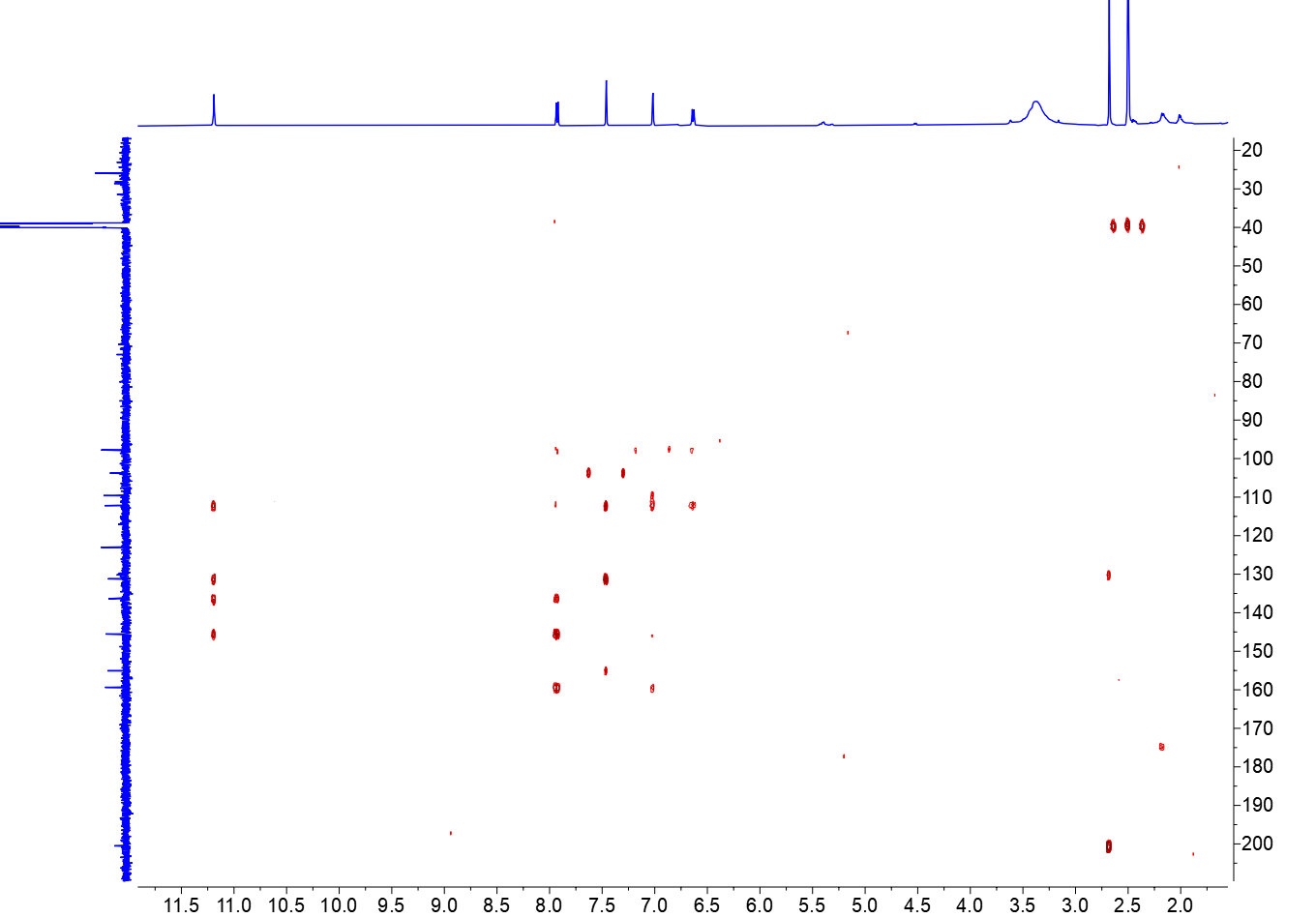


**Figure S5**. HMBC spectrum of compound **1**


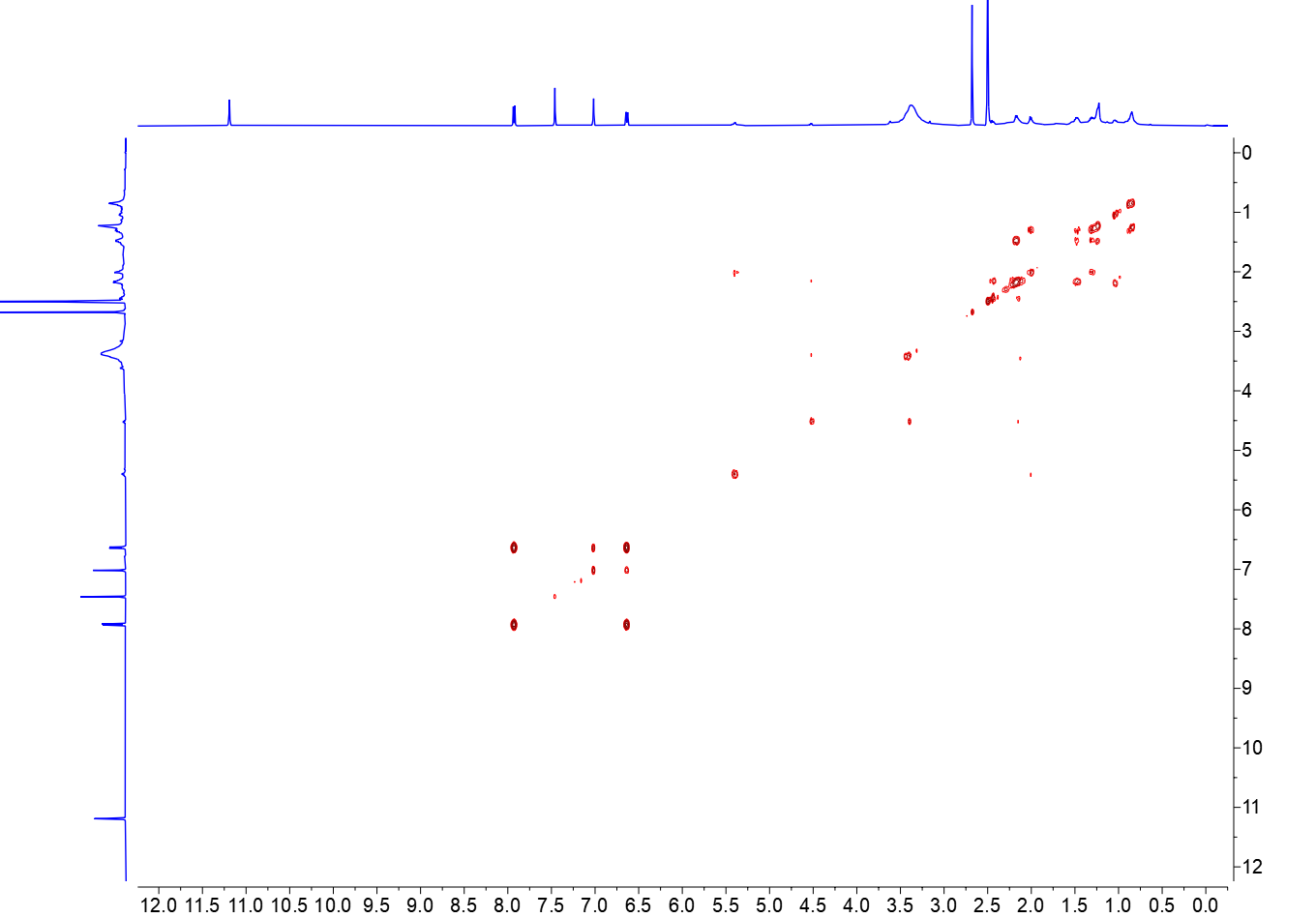


**Figure S6**. ^1^H-^1^H COSY spectrum of compound **1**

**Figure S7**. HR-ESI-MS of compound **2**


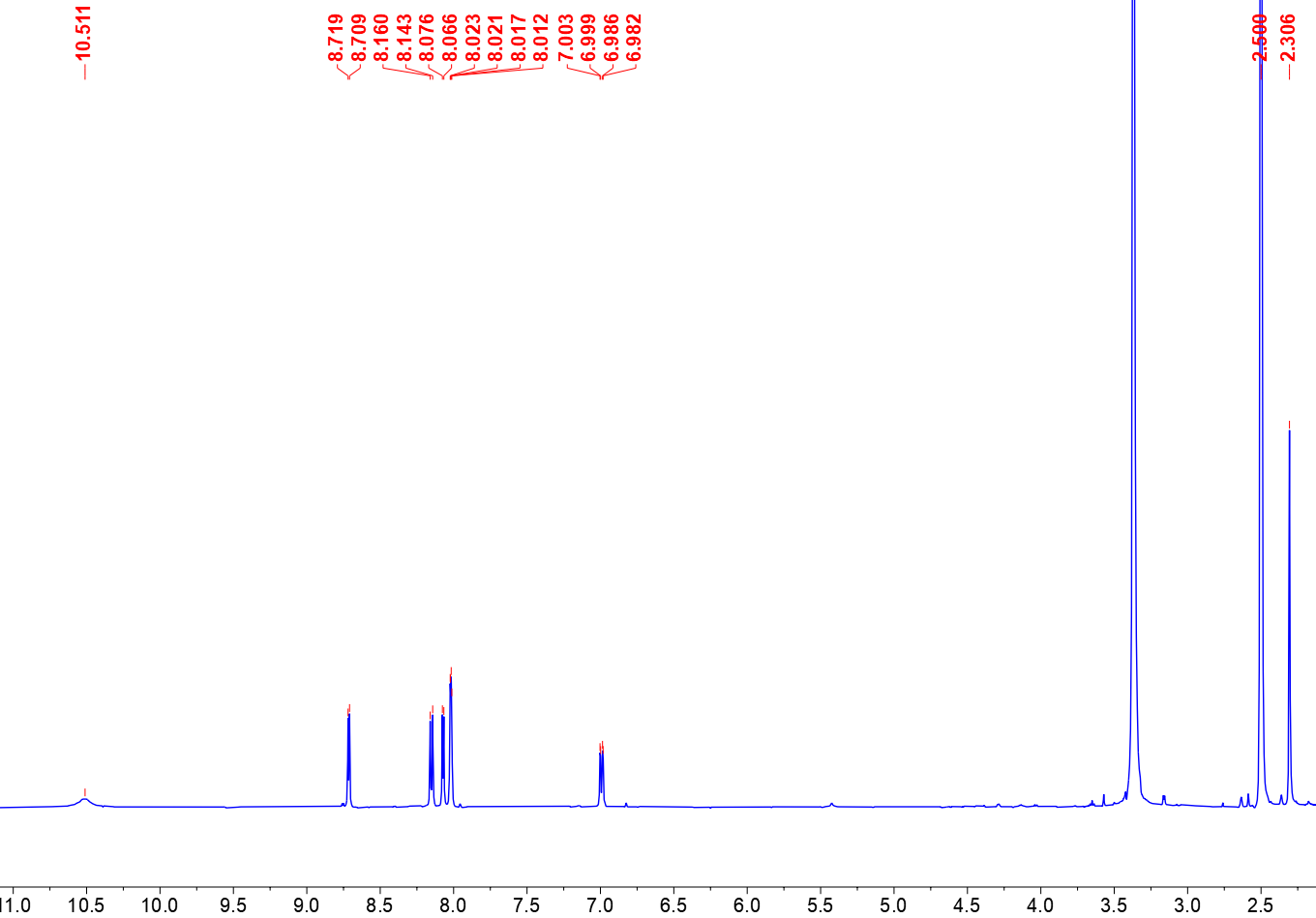


**Figure S8**. ^1^H-NMR spectrum of compound **2**


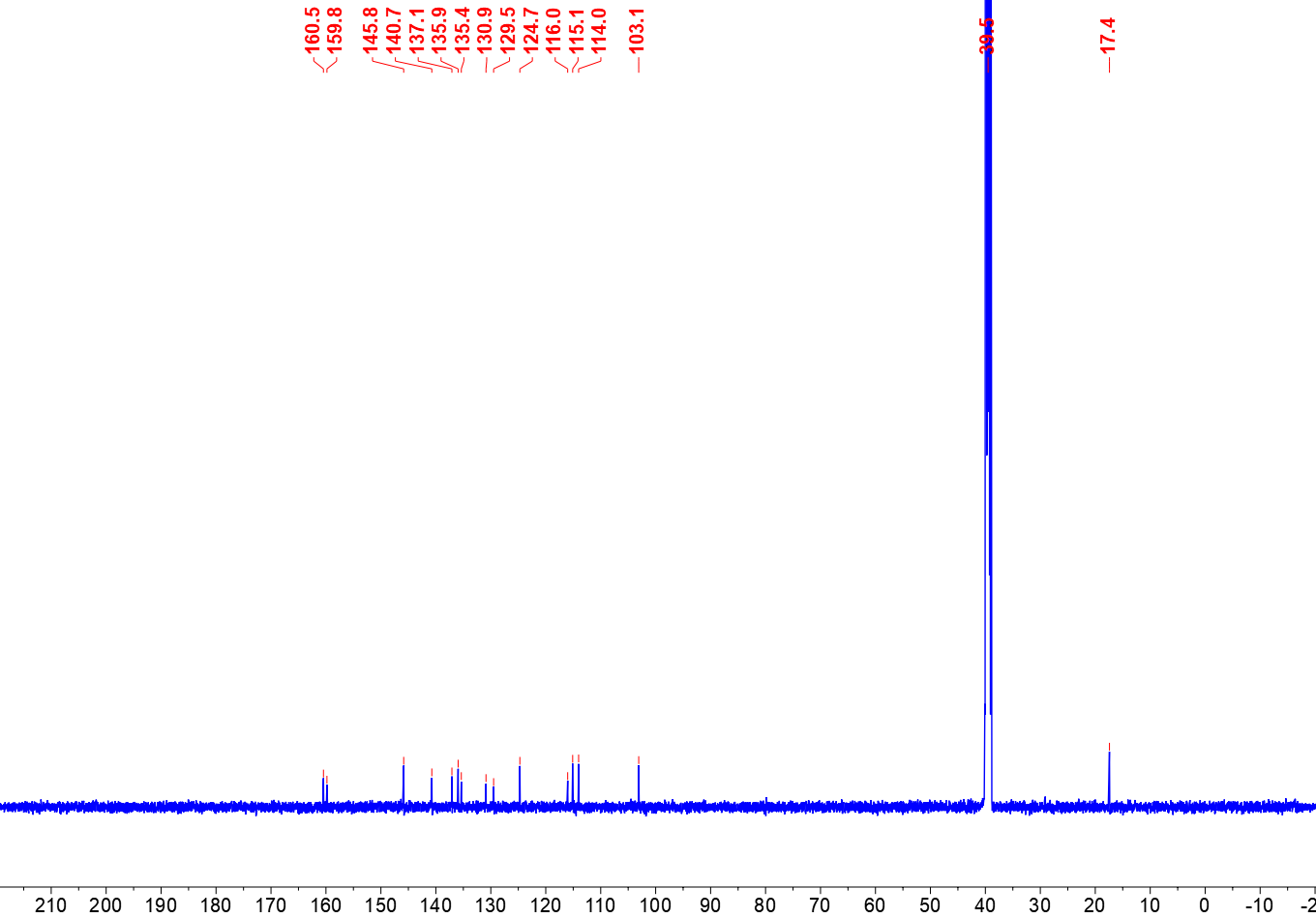


**Figure S9**. ^13^C-NMR spectrum of compound **2**


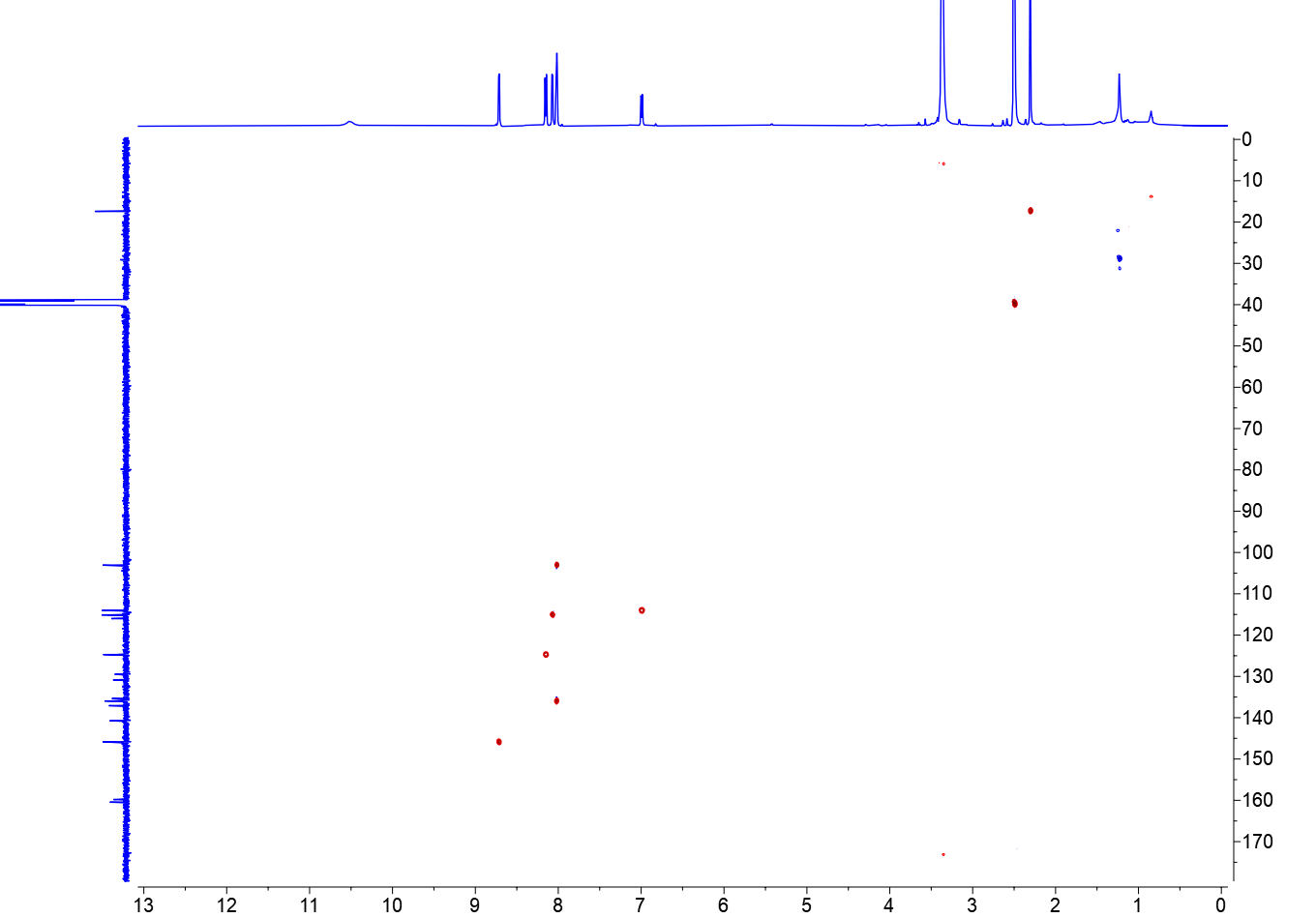


**Figure S10**. HSQC spectrum of compound **2**


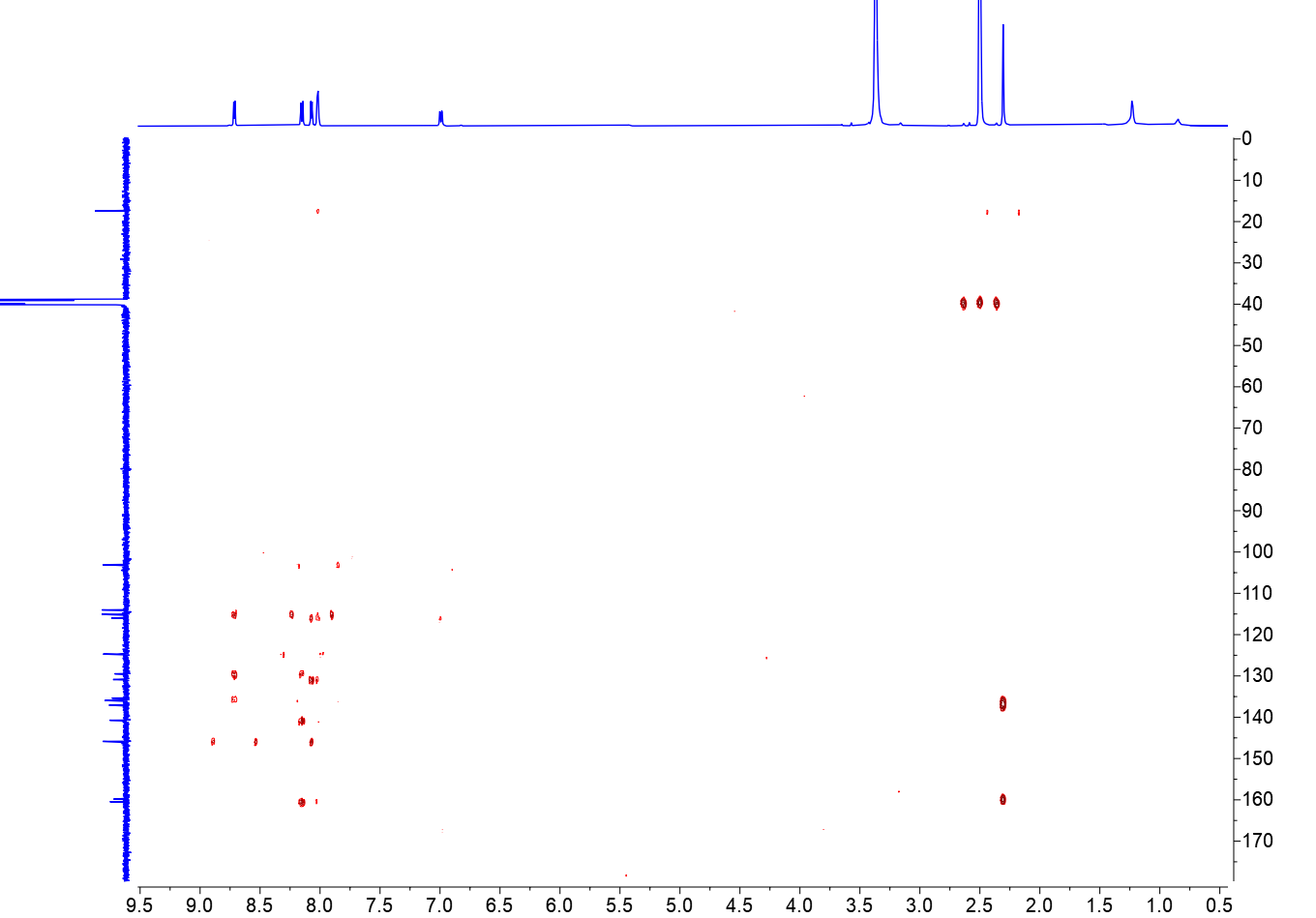


**Figure S11**. HMBC spectrum of compound **2**


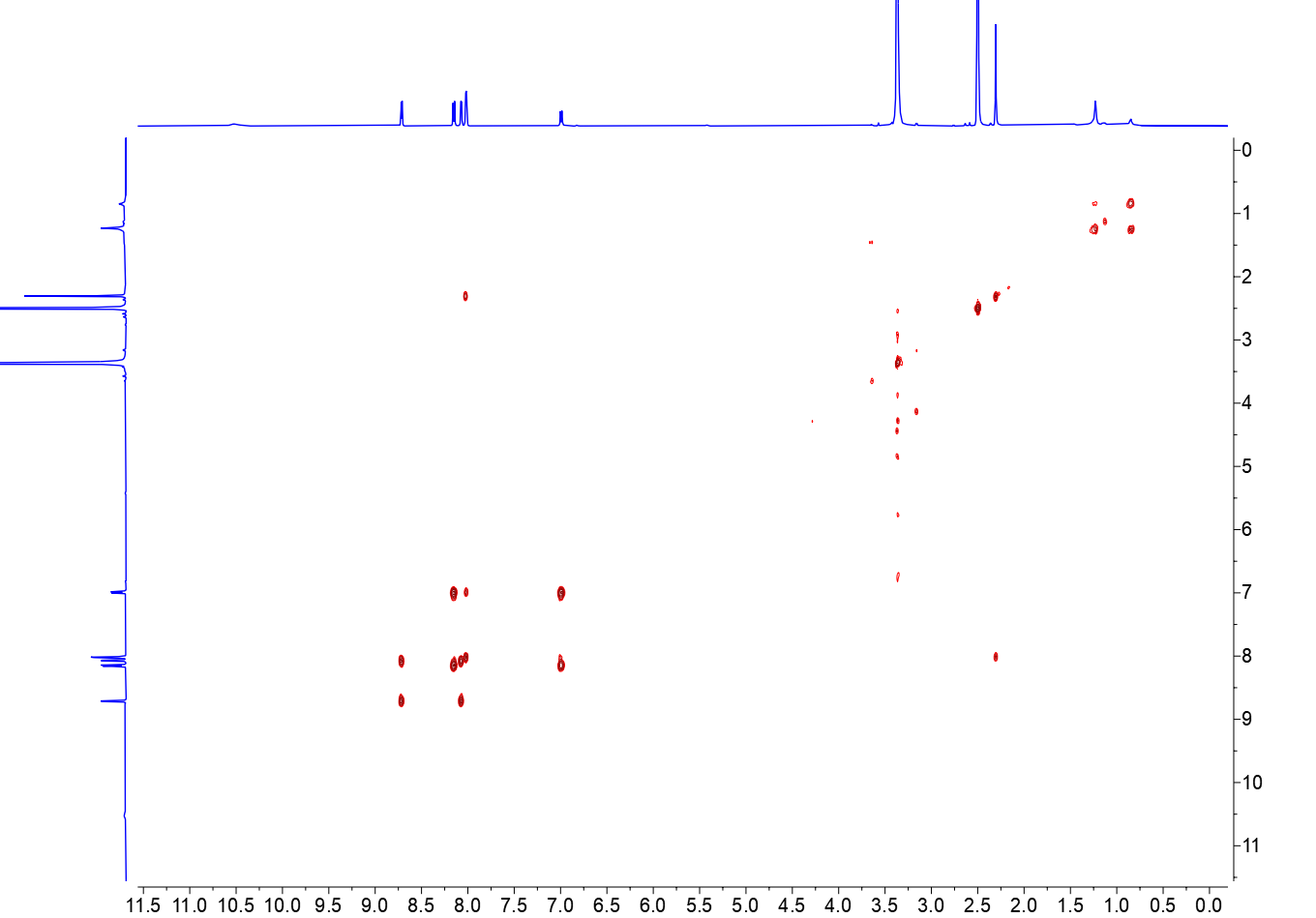


**Figure S12**. ^1^H-^1^H COSY spectrum of compound **2**

**Figure S13**. HR-ESI-MS of compound **3**


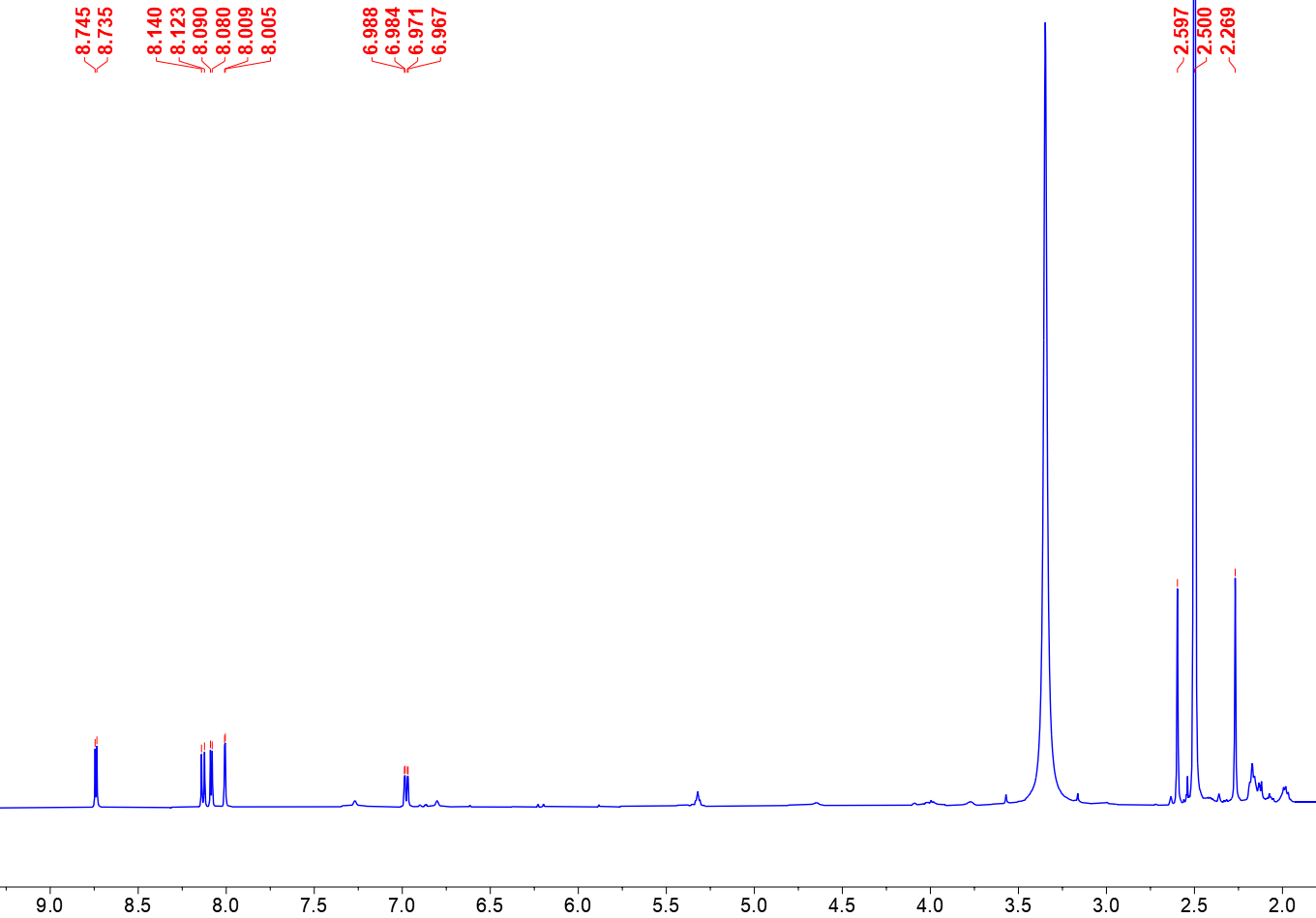


**Figure S14**. ^1^H-NMR spectrum of compound **3**


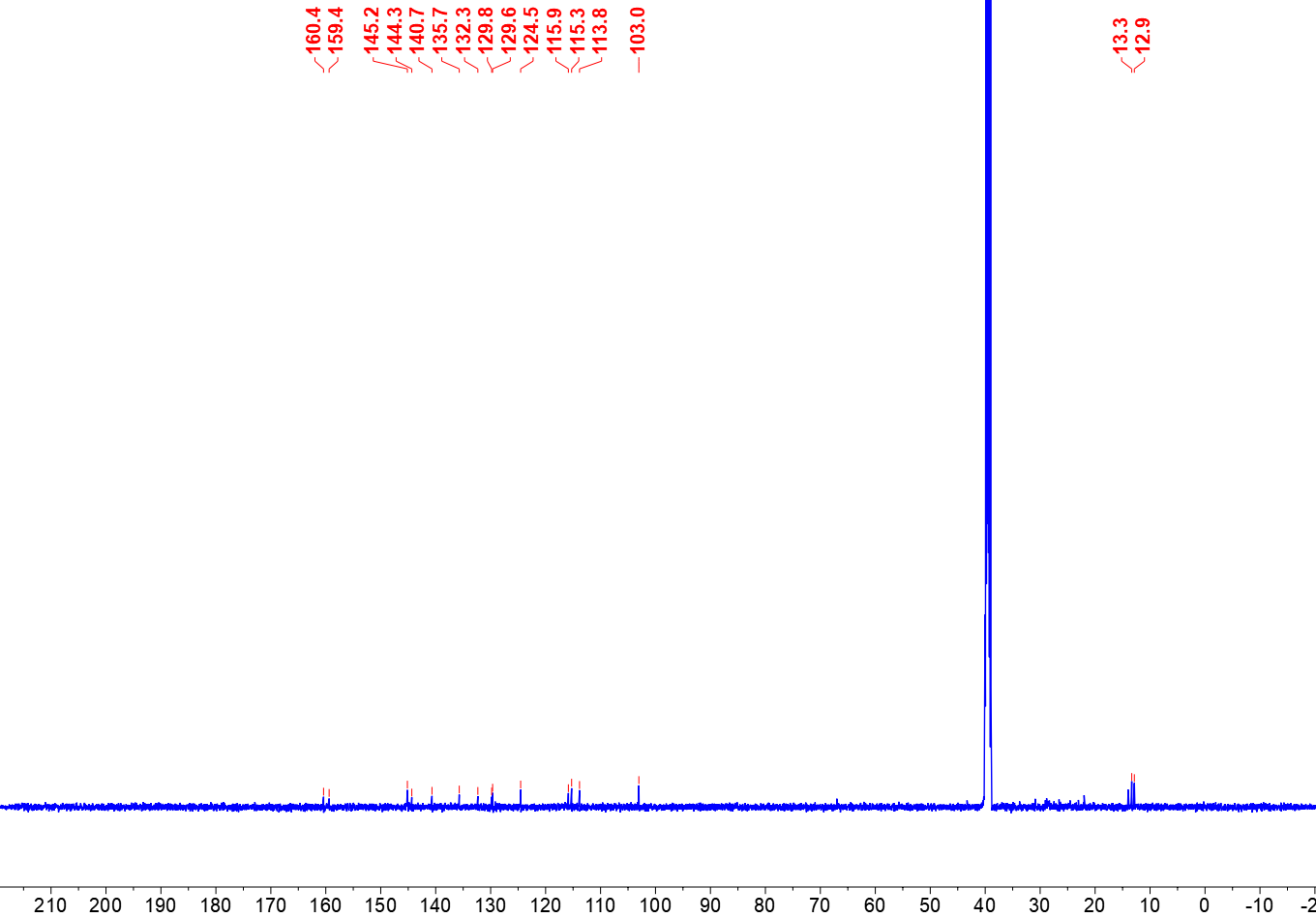


**Figure S15**. ^13^C-NMR spectrum of compound **3**


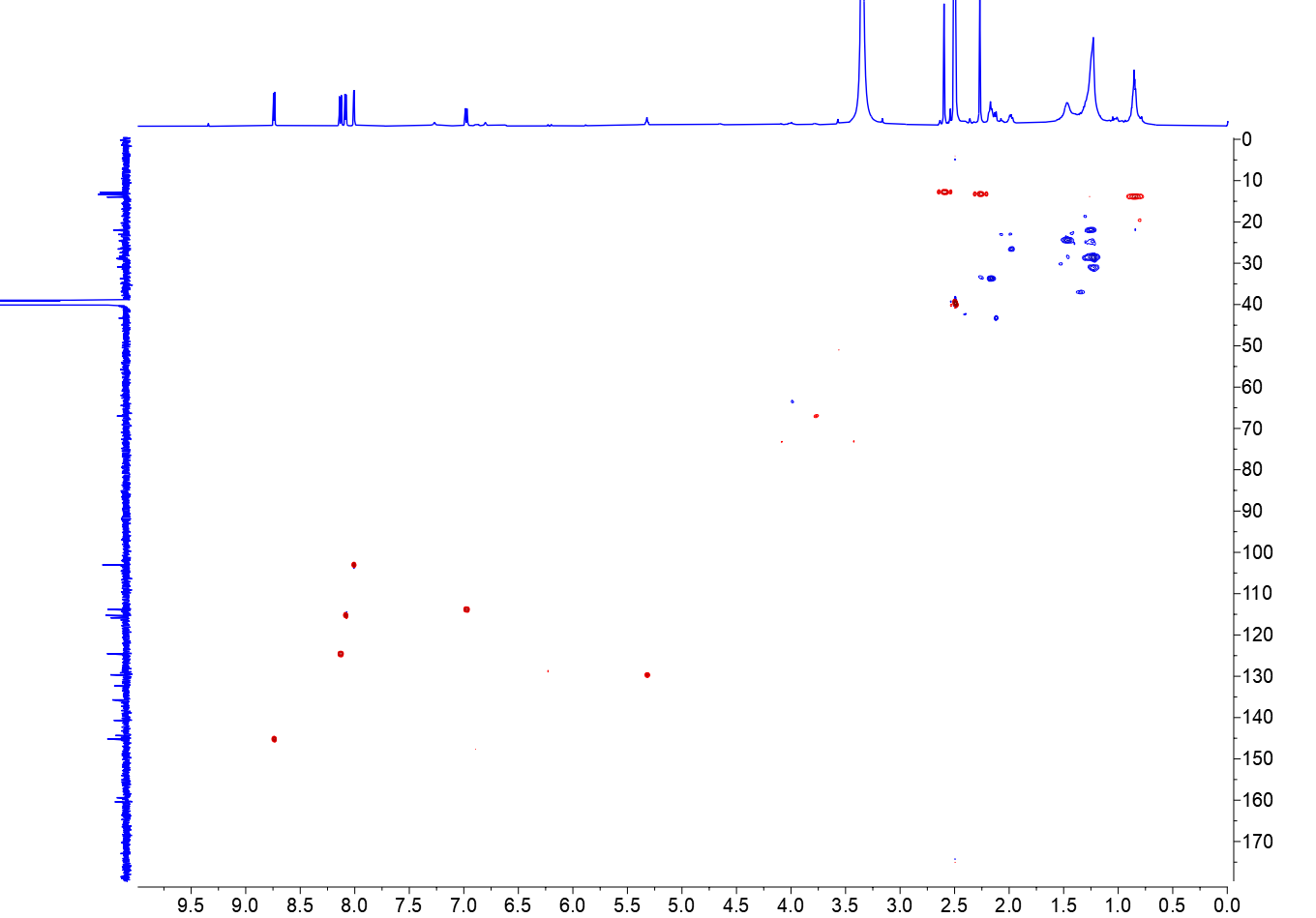


**Figure S16**. HSQC spectrum of compound **3**


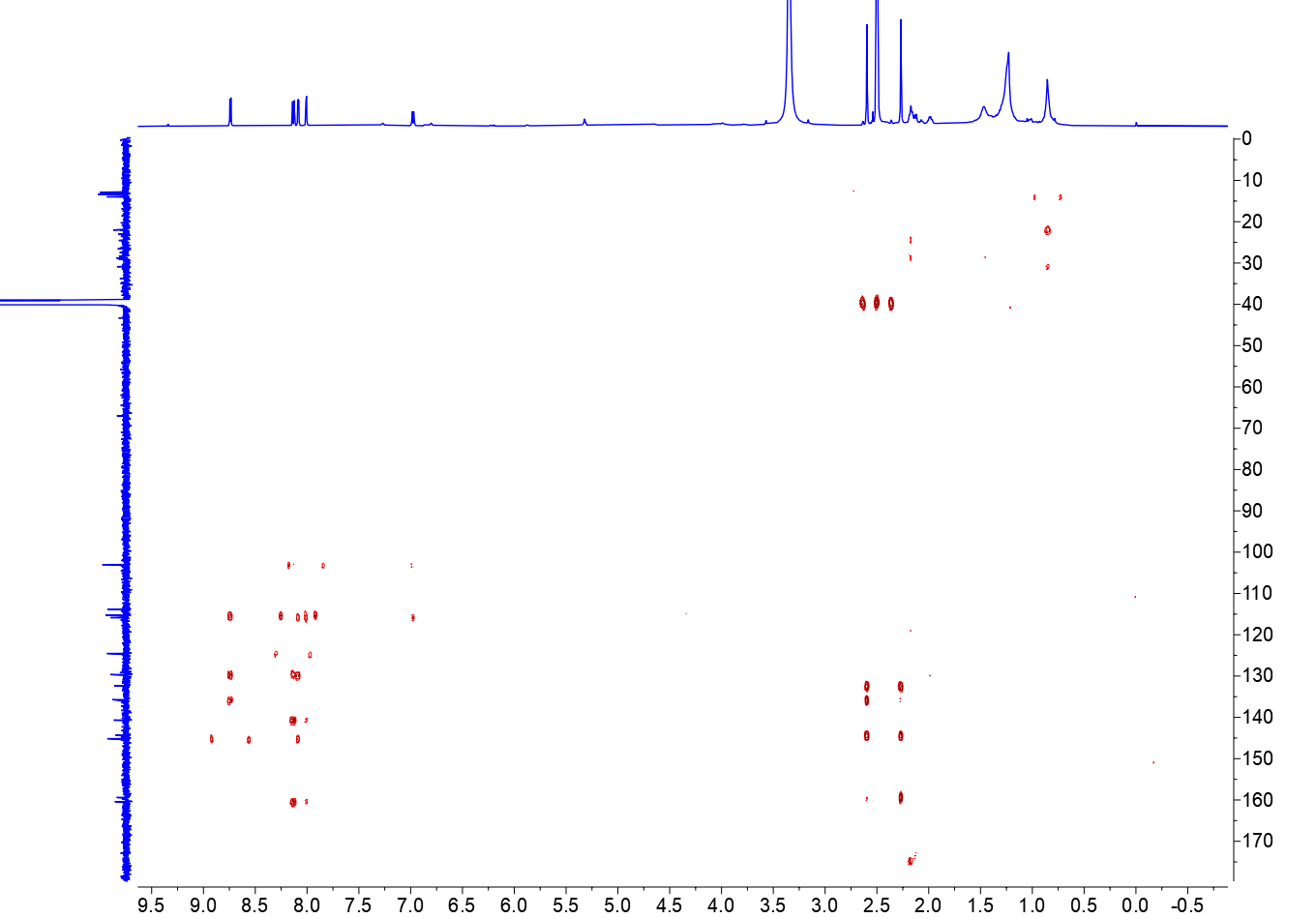


**Figure S17**. HMBC spectrum of compound **3**


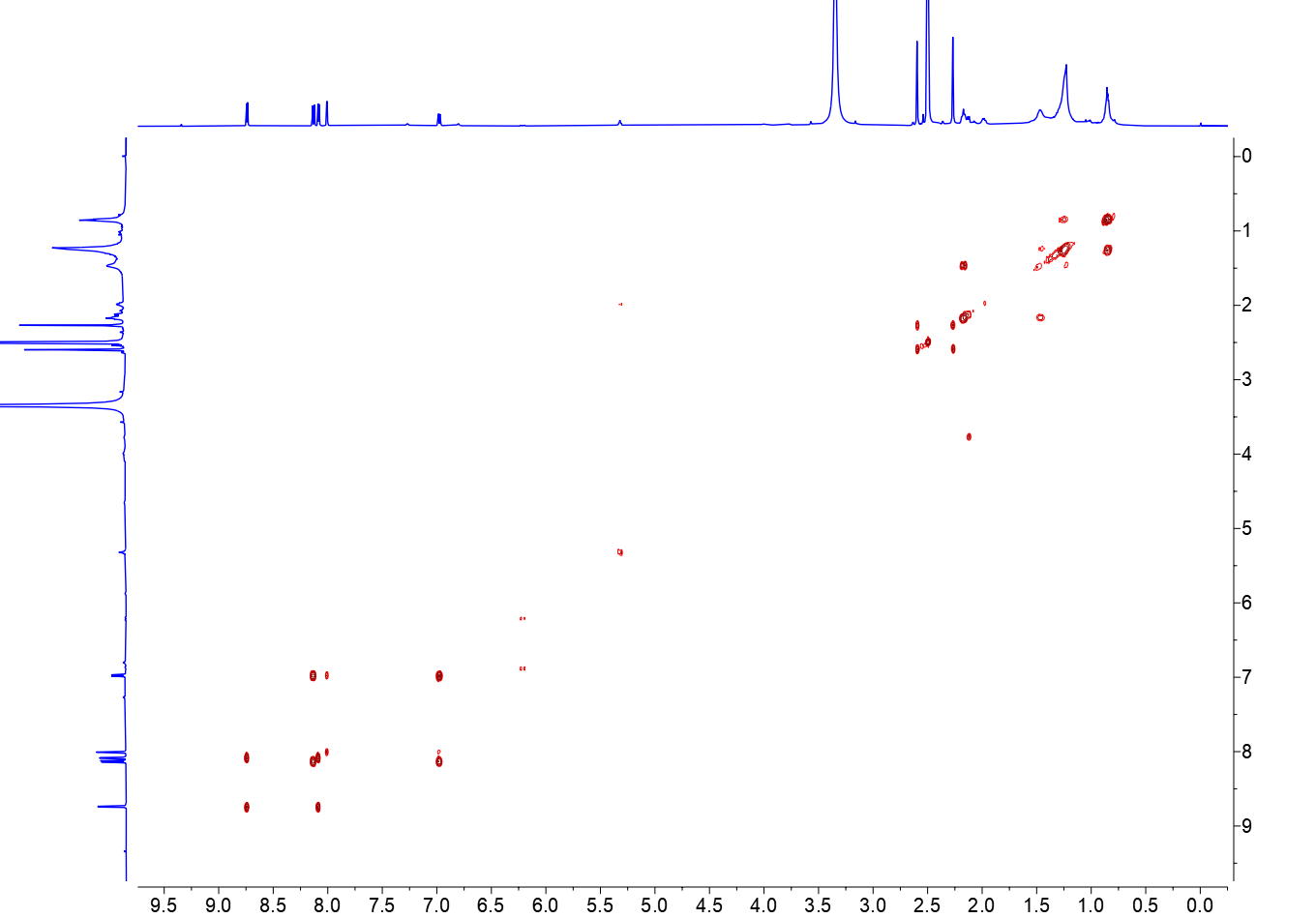


**Figure S18**. ^1^H-^1^H COSY spectrum of compound **3**
